# Osteogenesis imperfecta, intellectual disability and recurrent infections in a male with a pathogenic SASH3 variant

**DOI:** 10.1038/s41439-025-00323-1

**Published:** 2025-09-15

**Authors:** Jun Kido, Tomoyuki Mizukami, Yohei Misumi, Keishin Sugawara, Shouichirou Kusunoki, Naoto Nishimura, Takeshi Mizuguchi, Naomichi Matsumoto, Mitsuharu Ueda, Kimitoshi Nakamura

**Affiliations:** 1 Department of Pediatrics, Faculty of Life Sciences, Kumamoto University, Kumamoto, Japan; 2 Department of Pediatrics, Kumamoto University Hospital, Kumamoto, Japan; 3 Department of Pediatrics, National Hospital Organization Kumamoto Medical Center, Kumamoto, Japan; 4 Department of Neurology, Kumamoto University Hospital, Kumamoto, Japan; 5 Department of Human Genetics, Yokohama City University Graduate School of Medicine, Yokohama, Japan; 6 Department of Rare Disease Genomics, Yokohama City University Hospital, Yokohama, Japan; 7 Department of Clinical Genetics, Yokohama City University Hospital, Yokohama, Japan

**Keywords:** Severe combined immunodeficiency, Severe combined immunodeficiency

## Abstract

Src Homology 3 Domain-containing Adaptor Protein 3 (SASH3) deficiency is an X-linked immune disorder. Here we identified a male case with a pathogenic SASH3 variant (c.1039C>T [p.Arg347Cys]) who presented with osteogenesis imperfecta, intellectual disability and recurrent infections. While immunological features in this case were characterized, further studies are needed to determine the association between the SASH3 variant and the skeletal or neurological manifestations.

Src Homology 3 Domain-containing Adaptor Protein 3 (SASH3), also called SH3-domain-containing lymphocyte protein (SLY), is involved mainly in immune cell signaling and T cell activation. It functions as an adaptor protein that helps to regulate intracellular signaling pathways in the lymphocytes^1^. Delmonte et al.^2^ first reported that pathogenic variants of *SASH3* are associated with X-linked immune dysregulation disorders, and the disease concept is acquired. The *SASH3* gene was identified as the causative gene of immune dysregulation disorders in a study involving 1505 individuals from 1000 families with suspected or known inborn errors of immunity^3^. Here, we encountered a case of short stature due to osteogenesis imperfecta and intellectual disability. A pathogenic variant of *SASH3* was identified in the Initiative on Rare and Undiagnosed Diseases (IRUD)^4^, led by the Japan Agency for Medical Research and Development. Thus, we report the case of a male patient with SASH3 deficiency and discuss the clinical manifestations.

The patient was born via cesarean section at 39 weeks and 1 day of gestation. At birth, the patient’s height, weight and Apgar score were 46.0 cm (–1.5 standard deviation (s.d.)), 2556 g (–1.6 s.d.) and 9/10, respectively. The patient had no family history of hereditary disorders. The patient presented with a cleft lip and palate immediately after birth and hypertrophic pyloric stenosis. At the age of 2 years, the patient presented with a soft mass in the thenar region of the right thumb. Furthermore, the patient exhibited long bone dysplasia, reduced bone density, short stature, right renal agenesis and definite speech delay from early childhood (Fig. 1A–D). The patient’s developmental quotients (DQs) on the Kyoto Scale of Psychological Development at age 2 years and 8 months was 63 (total DQ) (postural-motor DQ, 54; cognitive-adaptive DQ, 70; and language-social DQ, 42). Between the ages of 5 and 7 years, the patient was hospitalized five times for infections such as bacterial pneumonia, bacterial cellulitis and rotavirus gastroenteritis. In each instance of hospitalization, the patient was admitted within 1 week and recovered with intravenous antibiotic therapy. Notably, despite the bacterial infections, the white blood cell counts at the time of admission remained low, ranging from 1300 to 5100/μl.

**Fig. 1:**
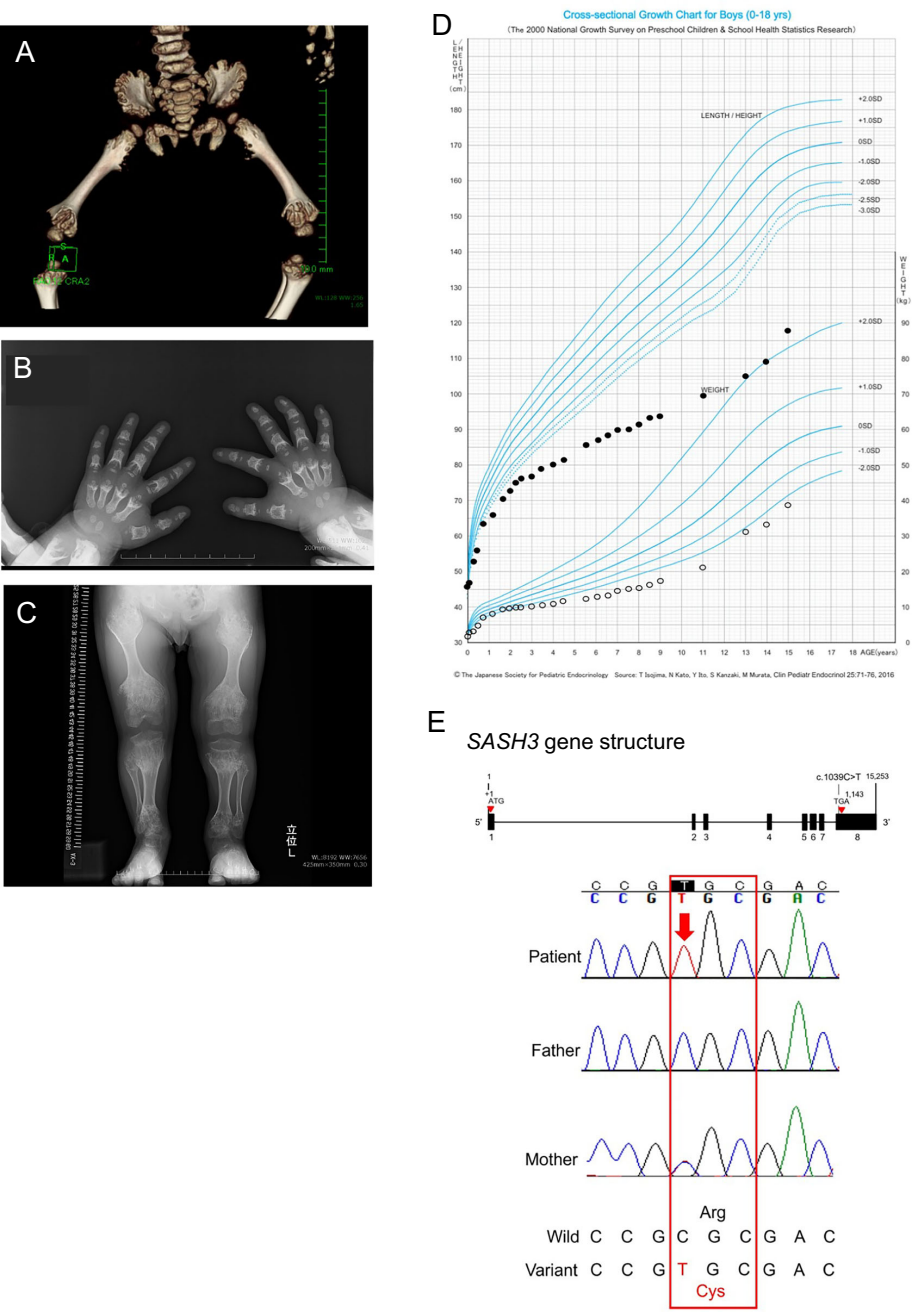
Abnormality of bone formation and lowered height and gene analysis in the patient. **A** Computed tomography at 1 year and 5 months of age. Bilateral hypoplasia of the diaphyseal ends of the femurs was observed. **B** X-ray of the hands at 2 years and 5 months of age. Underdevelopment of the finger bones in both hands was noted. **C** X-ray of the legs at 10 years of age. Shortening of the long bones and enlargement of the diaphyseal ends of the long bones are observed. **D** Growth curve. Marked growth retardation was observed in this patient. **E** Sanger sequencing. The patient has a hemizygous missense variant (c.1039C>T [p.Arg347Cys]) in the *SASH3* gene. This pathogenic variant was inherited from the mother, who carries the same variant in the heterozygous state.

At 14 years of age, the patient was diagnosed with intellectual disability (total intelligence quotient 67 on the Wechsler Intelligence Scale for Children III test), long bone dysplasia with impaired endochondral ossification, persistent short stature and hearing loss. Although the range of motion in the hip, knee and ankle joints is currently limited, the patient is able to walk independently.

The patient was registered with IRUD^3^, and whole-exome sequencing identified a hemizygous missense variant (c.1039C>T [p.Arg347Cys]) in *SASH3* (MIM *300441) (Fig. 1E and Supplementary Data 1). Since then, we investigated the patient’s immune function at 16 years of age (Fig. 2 and Supplementary Data 2). Laboratory tests revealed mildly decreased white blood cell counts. Immunoglobulin levels revealed IgG and IgA within the age-matched normal range, whereas IgM levels were decreased. Lymphocyte subset analysis showed a notable T cell reduction: CD3^+^ T cells accounted for 46.2%, CD4^+^ T cells for 15.2% and CD8^+^ T cells for 23.2%, indicating a substantial decrease in CD3^+^ and CD4^+^ T cells^5^, consistent with previous reports^2,6,7^ (Fig. 2). By contrast, CD19^+^ B cells comprised 24.2% and CD56^+^ natural killer (NK) cells 25.6%, with no decrease observed, differing from previously reported cases (Fig. 2). However, further analysis of B cells demonstrated a considerable reduction in IgM memory B cells (CD19^+^CD27^+^IgD^+^) at 1.6% and switched memory B cells (CD19^+^CD27^+^IgD^−^) at 2.4% (Fig. 2). Although the total B cell count and IgG levels did not decrease, these results indicated a clear impairment in B cell differentiation.

**Fig. 2:**
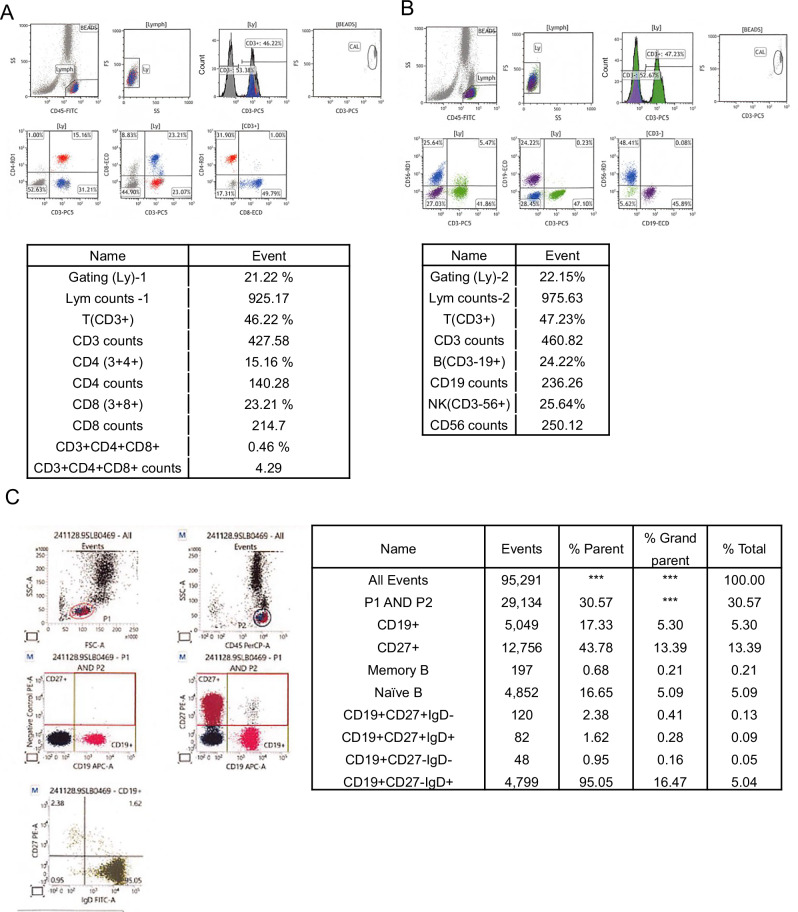
Immunophenotypic analysis of peripheral lymphocyte subpopulations in the patient. **A** Representative dot plots of T cell subpopulations, showing CD3, CD4 and CD8 expression profiles in peripheral blood lymphocytes **B** Representative dot plots of B and NK cell subpopulations, displaying CD3, CD19 and CD56 expression in peripheral blood lymphocytes. **C** Four-color flow cytometry analysis using IgD, CD27, CD45 and CD19 to evaluate B cell maturation and memory status. P1 and P2 denote the levels of all peripheral blood lymphocytes.

Thus, the patient was diagnosed with combined immunodeficiency owing to functional abnormalities in T and B cells. The immune function in this patient with SASH3 deficiency was generally consistent with the clinical features of SASH3 deficiency reported in previous studies^2,6,7^ (Supplementary Data 3). SASH3 deficiency is typically characterized by three main findings: cytopenia affecting one or more blood cell lineages (including white blood cells such as neutrophils and lymphocytes, red blood cells or platelets), hypogammaglobulinemia and reduced numbers of lymphocyte subsets (T cells, B cells or NK cells) (Supplementary Data 3).

This patient exhibited cytopenia and T cell reduction, consistent with prior findings. However, B cell counts remained within normal range. Although mild leukopenia was present, hypogammaglobulinemia was not observed, making immunodeficiency less clinically apparent. Moreover, this case had no history of recurrent or severe infections, including opportunistic infections, and the clinical course did not suggest overt immunodeficiency.

This case was conclusively diagnosed as SASH3 deficiency via IRUD; however, the disease was not suspected before undergoing IRUD. The patient’s primary clinical manifestations were osteogenesis imperfecta and intellectual disability.

According to Delmonte et al.^2^, patients with SASH3 deficiency demonstrate CD4^+^ T cell lymphopenia, reduced T cell proliferation, impaired cell cycle progression and elevated T cell apoptosis in response to mitogens. Moreover, five previously reported cases of SASH3 deficiency recapitulate several of the immunological defects described in two different Sly1-deficient mouse models (Sly1^Δ/Δ^ and Sly1^−/−^)^8,9^ (Supplementary Data 3). Specifically, (1) the absence of the SLY1 (SASH3) protein influences CD4^+^ T cell development, T cell proliferation and cytokine production. (2) SLY1 functions as an anti-apoptotic factor necessary for thymocyte development. (3) Antibody responses to T-dependent and T-independent antigens are impaired. Consequently, B cell differentiation and maturation are impaired.

So far, only four types of pathogenic variant (three nonsense variants: c.505C>T [p.Gln169*], c.733C>T [p.Arg245*] and c.862C>T [p.Arg288*], and one missense variant: c.1039C>T [p.Arg347Cys]) have been reported globally in patients with SASH3 deficiency (Supplementary Data 3). None of these variants is registered in the gnomAD^10^ or the Tohoku Medical Megabank^11^ genome databases. In silico analyses—including CADD (score: 25.0; deleterious), SIFT (deleterious), PolyPhen-2^12^ (probably damaging) and MutationTaster (deleterious)—consistently suggest that the c.1039C>T [p.Arg347Cys] variant is pathogenic. According to the guidelines of American College of Medical Genetics and Genomics and Association for Molecular Pathology, this variant is classified as likely pathogenic based on the criteria PS1, PM2 and PP3^13^.

Furthermore, Delmonte et al.^2^ investigated sequence motifs surrounding Arg347, a highly conserved residue from *Xenopus* to humans, and identified a putative protein-kinase-A-binding motif using a three-dimensional model of SASH3. The wild-type SASH3 protein has been shown to be phosphorylated at Ser349, supporting the functional significance of this motif. Therefore, the p.Arg347Cys amino acid substitution may impair SASH3 function by disrupting the Ser349 phosphorylation site, a key regulatory element.

Typically, the disease manifests in childhood^2,6^; however, diagnosis is often delayed. SASH3 deficiency may represent a mild or moderate form of this condition, although *SASH3* is the causative gene of combined immune deficiency. In addition, the unique clinical manifestations noted in this patient, metaphyseal dysplasia and intellectual impairment, have not been reported in all individuals with SASH3 deficiency. Whole-exome sequencing conducted using IRUD did not detect pathogenic variants in genes linked to osteogenesis imperfecta or intellectual disabilities. The genetic basis for these manifestations remains elusive.

In conclusion, we presented a case of SASH3 deficiency characterized by combined immune deficiency, metaphyseal dysplasia and intellectual impairment. Despite recurrent viral and bacterial infections during early life, the patient’s immune deficiency was evident from childhood but was not life-threatening.

## HGV Database

The relevant data from this Data Report are hosted at the Human Genome Variation Database at 10.6084/m9.figshare.hgv.3539.

## Supplementary information

Supplementary Data 1

Supplementary Data 2

Supplementary Data 3

## References

[1] Beer, S., Simins, A. B., Schuster, A. & Holzmann, B. Molecular cloning and characterization of a novel SH3 protein (SLY) preferentially expressed in lymphoid cells. *Biochim. Biophys. Acta*10.1016/S0167-4781(01)00242-1 (2001). 11470164 10.1016/s0167-4781(01)00242-1

[2] Delmonte, O. M., Bergerson, J., Kawai, T., Kuehn, H. S., McDermott, D. H., Cortese, I. et al. *SASH3* variants cause a novel form of X-linked combined immunodeficiency with immune dysregulation. *Blood*10.1182/blood.2020008629 (2021). 33876203 10.1182/blood.2020008629 PMC8462359

[3] Similuk, M. N., Yan, J., Ghosh, R., Oler, A. J., Franco, L. M., Setzer, M. R. et al. Clinical exome sequencing of 1000 families with complex immune phenotypes: Toward comprehensive genomic evaluations. *J. Allergy Clin. Immunol.*10.1016/j.jaci.2022.06.009 (2022). 35753512 10.1016/j.jaci.2022.06.009 PMC9547837

[4] Takahashi, Y. & Mizusawa, H. Initiative on rare and undiagnosed disease in Japan. *JMA J.*10.31662/jmaj.2021-0003 (2021). 33997444 10.31662/jmaj.2021-0003 PMC8119020

[5] Ding, Y., Zhou, L., Xia, Y., Wang, W., Wang, Y., Li, L. et al. Reference values for peripheral blood lymphocyte subsets of healthy children in China. *J. Allergy Clin. Immunol.*10.1016/j.jaci.2018.04.022 (2018). 29746882 10.1016/j.jaci.2018.04.022

[6] Labrador-Horrillo, M., Franco-Jarava, C., Garcia-Prat, M., Parra-Martínez, A., Antolín, M., Salgado-Perandrés, S. et al. Case report: X-Linked SASH3 deficiency presenting as a common variable immunodeficiency. *Front. Immunol.*10.3389/fimmu.2022.881206 (2022). 35464398 10.3389/fimmu.2022.881206 PMC9027814

[7] Novak, W., Berner, J., Svaton, M., Jimenez-Heredia, R., Segarra-Roca, A., Frohne, A. et al. Evans syndrome caused by a deleterious mutation affecting the adaptor protein SASH3. *Br. J. Haematol.*10.1111/bjh.19061 (2023). 37646304 10.1111/bjh.19061

[8] Beer, S., Scheikl, T., Reis, B., Hüser, N., Pfeffer, K., Holzmann et al. Impaired immune responses and prolonged allograft survival in Sly1 mutant mice. *Mol. Cell Biol.*10.1128/MCB.25.21.9646-9660.2005 (2005). 16227612 10.1128/MCB.25.21.9646-9660.2005 PMC1265838

[9] Reis, B., Pfeffer, K. & Beer-Hammer, S. The orphan adapter protein SLY1 as a novel anti-apoptotic protein required for thymocyte development. *BMC Immunol.*10.1186/1471-2172-10-38 (2009). 19604361 10.1186/1471-2172-10-38 PMC2717057

[10] Karczewski, K. J., Francioli, L. C., Tiao, G., Cummings, B. B., Alföldi, J., Wang, Q. et al. Genome Aggregation Database Consortium. The mutational constraint spectrum quantified from variation in 141,456 humans. *Nature*10.1038/s41586-020-2308-7 (2020). 32461654 10.1038/s41586-020-2308-7 PMC7334197

[11] Tadaka, S., Kawashima, J., Hishinuma, E., Saito, S., Okamura, Y., Otsuki, A. et al. jMorp: Japanese multi-omics reference panel update report 2023. *Nucleic Acids Res.*10.1093/nar/gkad978 (2024). 37930845 10.1093/nar/gkad978 PMC10767895

[12] Adzhubei, I. A., Schmidt, S., Peshkin, L., Ramensky, V. E., Gerasimova, A., Bork, P. et al. A method and server for predicting damaging messense mutations. *Nat. Methods*10.1038/nmeth0410-248 (2010). 20354512 10.1038/nmeth0410-248 PMC2855889

[13] Richards, S., Aziz, N., Bale, S., Bick, D., Das, S., Gastier-Foster, J. et al. Standards and guidelines for the interpretation of sequence variants: a joint consensus recommendation of the American College of Medical Genetics and Genomics and the Association for Molecular Pathology. *Genet. Med.*10.1038/gim.2015.30 (2015). 25741868 10.1038/gim.2015.30 PMC4544753

