# Supplementary material for: Osteogenesis imperfecta, intellectual disability and recurrent infections in a male with a pathogenic SASH3 variant
Source: Hum Genome Var. 2025 Sep 15;12:19. doi: 10.1038/s41439-025-00323-1 (PMC12434141; doi:10.1038/s41439-025-00323-1)
Supplement: Supplementary file 1 — Supplementary Data 1 [file 41439_2025_323_MOESM1_ESM.docx]

Supplemental Data 1 Information quality control report of the whole exome sequencing

A. Exome sequencing read coverage for protein coding sequences in this Family.

| Individual | Mean read depth | 5× (%) | 10× (%) | 20× (%) |
| --- | --- | --- | --- | --- |
| Patient | 67.19 | 98.3 | 98.1 | 97.6 |
| Father | 53.50 | 98.2 | 98.0 | 96.9 |
| Mother | 57.49 | 98.2 | 98.0 | 97.3 |

The mean depth of coverage against the RefSeq coding sequence was 53.50–67.19, and 98.0 %–98.1 % of the total coding sequences were covered by more than 10 reads.

B. Exome sequencing read coverage of *SASH3* in this Family.

| Individual | Mean read depth | 5× (%) | 10× (%) | 20×(%) |
| --- | --- | --- | --- | --- |
| Patient | 60.43 | 100.0 | 100.0 | 100.0 |
| Father | 39.79 | 100.0 | 100.0 | 99.0 |
| Mother | 88.05 | 100.0 | 100.0 | 100.0 |

A total of 100.0 % of the *SASH3* coding region was covered by more than 10 reads.
