# Supplementary material for: Osteogenesis imperfecta, intellectual disability and recurrent infections in a male with a pathogenic SASH3 variant
Source: Hum Genome Var. 2025 Sep 15;12:19. doi: 10.1038/s41439-025-00323-1 (PMC12434141; doi:10.1038/s41439-025-00323-1)
Supplement: Supplementary file 2 — Supplementary Data 2 [file 41439_2025_323_MOESM2_ESM.docx]

Supplemental Data 2. Laboratory data at age of 16 years in the patient

|  |  |  | Reference |
| --- | --- | --- | --- |
| Blood test | WBC  RBC  Hb  Hct  MCV  MCH  MCHC  RDW  Plate  Pct  MPV  PDW  Basophil  Eosinophil  Neutrophil  Lymphocytes  Monocytes  CD4 (%)  CD4  CD8 (%)  CD8  CD4/CD8  Basophil (Counts)  Eosinophil (Counts）  Neutrophil (Counts）  Lymphocytes（Counts）  Monocytes （Counts）  T-CD3  B-CD19  NK-CD56 | 3.0  5.88  16.5  47.8  81.3  28.1  34.5  13.2  261  0.24  9.2  9.6  0.3  8.4  48.5  37.4  5.4  18.8  194  21.6  224  0.87  0.0  0.2  1.4  1.1  0.1  49.7  28.9  19.7 | 3.3-8.6 x 10^3^/μL  4.35-5.55 10^6^/μL  13.7-16.8 g/dL  40.7-50.1 %  83.6-98.2 fL  27.5-33.2 pg  31.7-35.3 g/dL  12.0-14.2 %  158-348 103/μL  0.132-0.268 %  9.0-11.1 fL  0.2-1.4 %  0.4-8.6 %  42.4-75.0 %  18.2-47.7 %  3.3-9.0 %  %  /μL  %  /μL  x 10^3^/μL  x 10^3^/μL  x 10^3^/μL  x 10^3^/μL  x 10^3^/μL  %  %  % |
| [Immunoserological](https://eow.alc.co.jp/search?q=immunoserological&ref=awlj) [test](https://eow.alc.co.jp/search?q=test&ref=awlj) | IgG  IgA  IgM | 1298  230  29 | 861～1747 mg/dL  93～393 mg/dL  33～183 mg/dL |
| Lymphocyte stimulation test  (Phytohemagglutinin) | Stimulation  Non-Stimulation  Ratio (Stim/Non-Stim) | 92526  885  104.5 | cpm  cpm  147.5-1251.3 |
| Lymphocyte stimulation test  (Concanavalin A) | Stimulation  Non-Stimulation  Ratio (Stim/Non-Stim) | 67537  885  76.3 | cpm  cpm  38.1-385.5 |

Hb: hemoglobin, Hct: hematocrit, MCH: mean corpuscular hemoglobin, MCHC: mean corpuscular hemoglobin concentration, MCV: mean corpuscular volume, MPV: mean platelet volume, Pct: plateletcrit, PDW: platelet distribution width, Plate: platelet, RBC: red blood cell, RDW: red cell distribution width, WBC: white blood cell
