# Supplementary material for: Osteogenesis imperfecta, intellectual disability and recurrent infections in a male with a pathogenic SASH3 variant
Source: Hum Genome Var. 2025 Sep 15;12:19. doi: 10.1038/s41439-025-00323-1 (PMC12434141; doi:10.1038/s41439-025-00323-1)
Supplement: Supplementary file 3 — Supplementary Data 3 [file 41439_2025_323_MOESM3_ESM.docx]

Supplemental Data 3. Immunological character of 6 patients with SASH3 deficiency

|  | P1  (Delmonte et al)^2^ | P2  (Delmonte et al)^2^ | P3  (Delmonte et al)^2^ | P4  (Delmonte et al)^2^ | P5  (Labrador-Horrillo et al)^6^ | P6  This Case |
| --- | --- | --- | --- | --- | --- | --- |
| Sex | Male | Male | Male | Male | Male | Male |
| Age at diagnosis, years | 19 | 50 | 27 | 56 | 41 | 17 |
| Age at onset, years | 3 | 5 | 3 | N.A. | 4 | 5 |
| *SASH3* variants | p.R347C | p.R288* | p.R288* | p.R245* | p.Q169* | p.R347C |
| Laboratory data |  |  |  |  |  |  |
| Anemia | － | Yes | Yes | － | － | － |
| Neutropenia | Yes | Yes | Yes | － | － | Yes |
| Lymphopenia | － | Yes | － | Yes | － | Yes |
| Thrombocytopenia | Yes | － | Yes | － | － | － |
| IgG | Normal | IVIG | IVIG | **↓** | **↓** | Normal |
| IgM | **↓** | **↑** | **↓** | **↓** | **↓** | **↓** |
| IgA | Normal | **↓** | **↓** | **↓** | Normal | Normal |
| Vaccine | Yes | N.A. | N.A. | Yes | Yes | Yes |
| CD3+ T cells | Normal | **↓** | Normal | **↓** | Normal | **↓** |
| CD4+CD3+ T cells | **↓** | Normal | **↓** | **↓** | Normal | **↓** |
| CD8+CD3+ T cells | **↑** | **↓** | **↑** | Normal | Normal | Normal |
| CD19+ B cells | **↓** | **↓** | **↓** | **↓** | **↓** | Normal |
| CD3-CD56+ NK cells | **↓** | **↓** | **↓** | **↓** | Normal | Normal |

IVIG: Intravenous immunoglobulin therapy was needed. N.A.: not available

The vaccine was well tolerated in patients P1, P4, P5, and P6.
